# Dentistry as a Vocation for Profit, Especially in North Carolina

**Published:** 1902-04

**Authors:** B. F. Arrington

**Affiliations:** Goldsboro, N. C.


### DENTISTRY AS A VOCATION FOR PROFIT, ESPECIALLY IN NORTH CAROLINA.

BY DR. B. F. ARRINGTON, GOLDSBORO, N. C.

THE practice of dentistry is not as remunerative in North Carolina, and possibly in some other States, as it is generally supposed to be by a large majority of young men who select dentistry as a vocation for life study and service.

The time given and expenditure made in preparation requisite for the practice of dentistry with the majority of young men is unwisely appropriated, chiefly because so many, proportionately, are unsuited to professional life, and are better suited for many of the varied pursuits that present for their consideration, and from which larger gains could be obtained than from the practice of dentistry, especially that class of young men endowed with capacity to grasp and manipulate mechanical and business pursuits successfully.

The profession of dentistry as a profitable vocation has for some years past proved a decided failure in North Carolina. Owing to over-estimate, it is deceptive and allures to injury. The field is crowded, and the number of practitioners is increasing annually, greater than the demand justifies; consequently reduction of prices and curtailment of profits.

Many of the young men, captivated with the idea of preparing for the practice of dentistry, flattered with the prospect of professional life, are doubtless influenced and ruled principally by the supposition and conceived idea that there is easy life and big money in it, which is erroneous and seriously misleading, as many learn with sad regrets after years of practice with no bank account to draw upon to enable them to tide over comfortably in case of affliction and in old age.

The outlay in time and cash expenditure requisite in preparation and equipment for practice is greater now than some years back, and the full measure and status of educational requirements and expenditure is not yet definitely established, but it will in all probability be greater five years hence than at present, as there seems to be a growing disposition to make the term at dental colleges *four years instead of three,* and the State dental executive boards seem to be drawing the cords tighter every year, as if in effort to make it appear that the professors in dental colleges (educators) are not equal to a creditable and faithful performance of the duties of their daily lifework in training and preparing young men for the practice of dentistry. Rather assumptive and presumptive.

At present it requires from three to four years preparation and a cash outlay of from eighteen hundred to two thousand five hundred dollars for readiness to begin practice on a moderately equipped basis. Then comes a realization of facts, with disappointment and discontent in not realizing from annual practice several thousand dollars, as is the false anticipation of the majority. A seven or eight hundred dollar cash practice (not net) is about the maximum figures with the majority for some years, and with many during life practice. Truly a small amount, far short of anticipations, and is discouarging; but the presentation is correct, as many from sad experience in practice have learned and can attest.

There are now from three to four hundred practising dentists in North Carolina, who, as a whole, do not, I am sure, average an annual cash practice of one thousand dollars. Eight hundred would be a liberal estimate, possibly considerably in excess of average cash receipts. To say that there are not twenty dentists in the State whose average annual cash receipts from practice will not exceed two thousand five hundred dollars would not be underestimating. This makes quite a poor showing in a pecuniary point of view for a professional vocation that costs so much expenditure in preparation to commence practice. It is possible that a decade hence the discouraging features will be proportionately greater. The number of practitioners in the field will have increased largely, there will be more "dental parlors" of the "cheap John," underrating type open and bidding for patronage at low figures, and prices will be diminished below the level and range of an equitable and legitimate charge for skilful and meritorious service, a state of things possible and to be regretted, but inevitable unless there shall be wise management and successful legislation to prevent.

With the state of things existing as above stated, correct beyond questioning, the question may be asked, Wherein have the State dental executive boards in any way benefited the public, "the dear people" for whom they profess to feel such deep concern, elevated the profession, or in the slightest degree improved the practice of dentistry? No one can recognize beneficial results from their labors, and all must feel that if the executive boards are not the cause of the weakened and weakening state of the profession, they certainly have not proved a preventive, therefore are useless and not further needed, and will, if continued, from this on tend to evil more than good, as the tendency of their existence will be to weaken and lower the importance and dignity of the profession in the general estimation of the public not only in North Carolina, but in other States, by creating false impressions relative to instruction and work in dental colleges.

Whenever we ignore the professional ability, the high status of attainment, and the able work performed by dental college professors in the discharge of their faithful lifework in educating and preparing young men for the practice of dentistry, and educate the public, as dental executive boards are doing, to regard the work in college on the line of instruction and preparation defective and a failure to the extent that State executive boards are requisite to pass upon and correct it, the whole fabric weakens in public estimation and must fail of right appreciation and usefulness.

When rightly considered and thoroughly analyzed as an organized protection for the public, and for professional uplifting and superior excellence and advancement, the whole thing (executive boards) presents an amusing and farcical aspect, and evidences most decidedly a weak feature in professional work. Like other crazes, it will have its run and may die of its own weakness and need of props to sustain.

We have good men of the profession on the State executive board in North Carolina (as it is we will hope in other States), but they are not superior in educational advantages, professional attainments, and practical skill to many dentists practising in the State. To credit them with ability and attainments equal to and superior to that of faculties in dental colleges to examine and pass upon the merits and qualifications of aspirants for practice would be erroneous and absurd in the extreme. Let the boasted pretensions be what they may, there is no such advanced attainments in the ranks of the profession in North Carolina at present, and possibly never will be.

Considering the many and varied avenues of industry and creditable pursuits now open and presenting to young men in all sections of our country, it is surprising that so many (some utterly unsuited) take to and embark in the practice of dentistry when it promises such poor compensation, knowing, too, that after expenditure of time and money, outfit procured, and diploma in hand, they must then confront the State executive boards and submit to the trying ordeal (legal, but useless) of testing the competency and work of dental college faculties, and to determine if a gracious permit in the shape of a license to practice shall be granted. Thoughtless, reckless, and unwise.

Young men giving thought and study to the subject of dentistry, with a view to practising in North Carolina, are admonished to consider and investigate carefully before deciding to embark in a line of lifework that may prove disappointing and produce fruit more bitter than sweet, as has been the experience of many who were easily deceived and tempted to embark hastily and without due reflection, as many are now doing, and to regret at leisure in after years that they had not more wisely measured and counted cost, and appropriated means, talent, and energy in a different direction, for the good of self and of State and country.

We have at present fifty or more dental colleges, variously located from Maine to Texas. Unquestionably too many for the credit of the profession and good of the public. If all could be closed but a dozen or so of the best type, with true stamp of excellence, for the next ten years, to meet the requisite demands only, it would be better for the profession and the people. Abuse and injury often comes from overcrowding. There is such a thing as surfeiting with too much of a good thing, as is evidenced in the rapid increase of dental colleges and the number of graduates annually turned out. The supply, such as it is, in some instances is vastly greater than the demand, consequently it works evil to the profession and evil to the public. When we have learned to demonstrate and practise more appreciation of quality than quantity a better state of things will exist. There will be fewer dental colleges, but better colleges as a whole, fewer annual graduates proportionately, but of better type and more thoroughly prepared to commence practice without State check or hinderance. Dental executive boards and dental parlors, one about as much needed as the other and both hurtful to the best interests of the profession, would soon vanish, and be recollected only as things of the past. The future of dentistry as a profession will be more promising, the public will be better served and compensation for service rendered will be more generous and universally liberal and satisfactory, and the temptation, for many reasons, will be more inviting and encouraging to study and prepare for the practise of dentistry than at the present time.

It may be a decade or several decades before the desired and needed benefits through reform can be effected, but it is only a question of reasonable time if right action and harmonious effort on the part of all who should feel interested for the change shall be persevered in. In the mean time there are and will be many opportunities and broad fields of labor presented for the choice and free exercise of brain and muscle of ambitious young men that had better look to other pursuits (more profitable) to which they are by nature and cultivation better suited than the practice of dentistry. Such are established, candid convictions after watchful observance of the progress and retrogression of dentistry, with varying features of change, favorable and unfavorable in theory and practice, for a period of many years, more than half a century.

